# Targeting Thyroid Cancer Microenvironment: Basic Research and Clinical Applications

**DOI:** 10.3389/fendo.2013.00167

**Published:** 2013-11-04

**Authors:** Carmelo Nucera

**Affiliations:** ^1^Human Thyroid Cancers Preclinical and Translational Research Laboratory, Experimental Division of Cancer Biology and Angiogenesis, Department of Pathology, Beth Israel Deaconess Medical Center, Simon C. Fireman Research Center, Harvard Medical School, Boston, MA, USA

**Keywords:** thyroid cancer, microenvironment, BRAF^V600E^, translational, targeted therapy, biomarkers, extracellular matrix, mouse models

## Introduction

Thyroid cancer is the most common endocrine malignancy and its incidence has increased considerably over the past few decades (1). This Research Topic is devoted to understanding the molecular mechanism of human thyroid cancer, with an emphasis on translation to the clinic. The original research papers assembled here probe the pathogenetic pathway from a wide range of approaches, including: (1) the role of microenvironment in thyroid cancer, (2) the role of deiodinases and epigenes leading to thyroid cancer, (3) functional studies of genetic mutations that shed insights into the etiology and pathogenesis of papillary thyroid micro-carcinoma or carcinoma (PTC), (4) genome-wide studies that describe patho-physiological mechanisms for thyroid cancer, and (5) epidemiological studies of thyroid cancer incidence and (6) the influence of environmental factors on development of human PTC.

## Scope

The articles gathered here reflect a particular interest in the genetic and epigenetic factors that could affect the thyroid cancer cell and its microenvironment. In particular, this topic will present preclinical and clinical research addressing the following issues: (i) identification of specific molecular patterns of thyroid tumorigenesis and tumor microenvironment, which could suggest new directions in pharmacotherapy research; (ii) discovery of new biomarkers to predict response or resistance to drug treatment, facilitating targeted cancer therapies and patient follow-up after treatment; (iii) discovery of environmental risk factors that might affect PTC development; and (iv) evaluation of preclinical models of human thyroid cancers with regard to their suitability for testing new drugs and molecule-targeted agents and for studies of targetable mechanisms of oncogenesis, malignant progression, and metastatic disease.

Specific areas of investigation in this topic include the following as they relate to thyroid cancer: epidemiology and clinical research; epigenetics in thyroid; personalized medicine in thyroid; the tumor microenvironment; novel applications of bioinformatics in human thyroid cancer cell lines; molecular characterization of thyroid tumors; and use of diagnostic and prognostic biomarkers.

## Why is Targeting the Thyroid Carcinoma Microenvironment Translational?

Currently no successful treatment is available for advanced thyroid cancer, including poorly differentiated (PDTC), anaplastic/undifferentiated (ATC), and metastatic recurrent/persistent differentiated PTC that is not responsive to radio-iodine therapy.

The past decade of thyroid cancer research has yielded fundamental advances with profound translational potential. Identification of molecular markers, including oncogenes (e.g., the BRAF^V600E^ mutation), tumor suppressors (e.g., TP53 mutations), and translocations (e.g., RET/PTC, PAX8/PPARγ), have clarified molecular mechanisms underlying the pathogenesis of thyroid cancer. However, correlations between each biomarker and prognosis/outcome have not yet been determined in a broad cohort of patients with metastatic thyroid cancer. Currently, BRAF^V600E^ is the most frequent genetic hallmark of PTC and has been highlighted as a prognostic biomarker to improve risk stratification of patients with PTC, including low risk PTC (2–7). Recently, a retrospective multicenter study has also shown that the occurrence of the BRAF^V600E^ mutation was significantly associated with increased cancer-related mortality among patients with PTC. However, because mortality in patients with PTC was low and the association was not independent of PTC clinico-pathologic features, the role of BRAF^V600E^ as marker of mortality risk in patients with PTC remains to be determined (3).

Preclinical studies have shown that PTC carrying the BRAF^V600E^ mutation are dependent on this oncoprotein for viability; both genetic and pharmacological inhibition of BRAF^V600E^ expression or activity is associated with thyroid carcinoma regression and restoration of radio-iodine uptake *in vivo* in mice (8). Furthermore, BRAF^V600E^ plays an important role in PTC progression to ATC through genes fundamental in the regulation of tumor microenvironment by triggering tumor invasion and metastasis (9). Therefore, testing a patient’s thyroid cancer for BRAF^V600E^ will yield important information about potential tumor aggressiveness and inform future use of targeted therapies with selective BRAF^V600E^ inhibitors. Collectively, these findings suggest a potential translational application for anti-BRAF^V600E^ therapy in clinical trials for patients with thyroid cancers refractory to radio-iodine treatment and surgically inoperable thyroid cancers.

BRAF^V600E^ affects the expression of tumor extracellular matrix (ECM) non-cellular components [e.g., Thrombospondin-1 (TSP-1), integrins and others] (9) by regulating PTC cell microenvironment communications; indeed, the molecular action of BRAF^V600E^ appears to affect both the migratory and invasive properties of the human thyroid cancer cell itself (10), as well as other cell types of the thyroid tumor microenvironment. Knowledge about new BRAF^V600E^-dependent targets (11) may help identify secreted factors that could serve as novel prognostic biomarkers and/or innovative therapeutic strategies in BRAF^V600E^-positive human thyroid cancers. For example, tumor-associated lymphocytes and high FoxP3(+) regulatory T cell (Treg) frequency in primary PTC correlates with more aggressive disease (12, 13). This suggests Treg frequency could be a predictive factor in PTC and that the suppressive effects of Treg could be considered in the design of immune-based therapies in PTC. Also, Ryder et al. found that tumor-associated macrophages (TAMs) may facilitate thyroid cancer progression (14), showing that the presence of a high density of TAMs in advanced metastatic thyroid cancers correlates with invasion and decreased cancer-related survival.

In summary, novel therapeutic strategies that target the metastatic thyroid carcinoma microenvironment (i.e., ECM cellular and non-cellular components) could offer an additional approach to the treatment of patients with these types of cancers. Targeting other cell types in the microenvironment instead of, or in addition to, the BRAF^V600E^-positive metastatic thyroid cancer cell might also minimize anti-BRAF^V600E^ drug resistance and provide potential additional therapeutic benefits. Determining the effects of factors in the thyroid tumor microenvironment will further define the spectrum of molecular mechanisms underlying signaling in metastatic thyroid carcinomas. Understanding the extent to which microenvironment factors participate in the aberrant behavior of BRAF^V600E^-positive human metastatic thyroid cancer cells will reveal whether the microenvironment is a promising target for development of new therapies. In summary, current research suggests that novel therapies directed against “microenvironment-specific targets” of thyroid carcinoma are a promising approach and should be developed and tested in preclinical/translational models of human metastatic thyroid cancer in the near future.
